# Acute-Phase Levels of CXCL8 as Risk Factor for Chronic Arthralgia Following Chikungunya Virus Infection

**DOI:** 10.3389/fimmu.2021.744183

**Published:** 2021-10-01

**Authors:** Leile Camila Jacob-Nascimento, Caroline Xavier Carvalho, Monaíse Madalena Oliveira Silva, Mariana Kikuti, Rosângela Oliveira Anjos, Jordana Rodrigues Barbosa Fradico, Ana Carolina Campi-Azevedo, Laura Beatriz Tauro, Gúbio Soares Campos, Patricia Sousa dos Santos Moreira, Moyra Machado Portilho, Olindo Assis Martins-Filho, Guilherme Sousa Ribeiro, Mitermayer Galvão Reis

**Affiliations:** ^1^ Instituto Gonçalo Moniz, Fundação Oswaldo Cruz, Salvador, Brazil; ^2^ Instituto Oswaldo Cruz, Fundação Oswaldo Cruz, Rio de Janeiro, Brazil; ^3^ Instituto de Saúde Coletiva, Universidade Federal da Bahia, Salvador, Brazil; ^4^ Instituto René Rachou, Fundação Oswaldo Cruz, Belo Horizonte, Brazil; ^5^ Instituto de Biologia Subtropical, Consejo Nacional de Investigaciones Científicas y Tecnicas - Universidad Nacional de Misiones, Puerto Iguazú, Argentina; ^6^ Instituto de Ciências da Saúde, Universidade Federal da Bahia, Salvador, Brazil; ^7^ Faculdade de Medicina da Bahia, Universidade Federal da Bahia, Salvador, Brazil; ^8^ Yale School of Public Health, Yale University, New Haven, CT, United States

**Keywords:** chronic arthralgia, chemokines, cytokines, chikungunya, serum biomarkers, cohort

## Abstract

The immunopathogenesis of chikungunya virus (CHIKV) infection and the role of acute-phase immune response on joint pain persistence is not fully understood. We investigated the profile of serum chemokine and cytokine in CHIKV-infected patients with acute disease, compared the levels of these biomarkers to those of patients with other acute febrile diseases (OAFD) and healthy controls (HC), and evaluated their role as predictors of chronic arthralgia development. Chemokines and cytokines were measured by flow Cytometric Bead Array. Patients with CHIKV infection were further categorized according to duration of arthralgia (≤ 3 months *vs >*3 months), presence of anti-CHIKV IgM at acute-phase sample, and number of days of symptoms at sample collection (1 *vs* 2-3 *vs* ≥4). Patients with acute CHIKV infection had significantly higher levels of CXCL8, CCL2, CXCL9, CCL5, CXCL10, IL-1β, IL-6, IL-12, and IL-10 as compared to HC. CCL2, CCL5, and CXCL10 levels were also significantly higher in patients with CHIKV infection compared to patients with OAFD. Patients whose arthralgia lasted > 3 months had increased CXCL8 levels compared to patients whose arthralgia did not (p<0.05). Multivariable analyses further indicated that high levels of CXCL8 and female sex were associated with arthralgia lasting >3 months. Patients with chikungunya and OAFD had similar cytokine kinetics for IL-1β, IL-12, TNF, IFN-γ, IL-2, and IL-4, although the levels were lower for CHIKV patients. This study suggests that chemokines may have an important role in the immunopathogenesis of chronic chikungunya-related arthralgia.

## Introduction

In the last decade, chikungunya virus (CHIKV) has caused several outbreaks worldwide and became a serious public health issue in the South and Central Americas, mainly in Brazil (1, 2). The first autochthonous cases of CHIKV infection in Brazil were reported in September 2014, in the city of Oiapoque, and in the city of Feira de Santana, States of Amapá and Bahia, respectively (3–5). Retrospectively, we found that the virus was also circulating in Salvador, the capital of Bahia, in September 2014 (6).

Acute CHIKV infection in humans may cause mild to moderate febrile disease, which is typically accompanied by rash, headache, myalgia, and intense polyarthralgia. Although most of the patients have a self-limited disease course, some individuals can progress to a chronic phase, characterized by continuous of recurring polyarthralgia that may last from months to years (7). Chronic arthralgia post-CHIKV infection also can lead to limitation of movements, incapacity for work, and even depression, directly affecting household incomes and representing a significant disease burden for affected populations (8–10). Therefore, it is necessary to elucidate the mechanisms that lead to chronic articular pain in some patients with chikungunya in order to prevent this complication.

Studies to determine the mechanisms of arthropathy associated with CHIKV infection have highlighted the role of the immune system in the pathogenesis of the disease (11). It has been described that a robust cytokine innate immune response can control CHIKV infection and protect against chronic arthropathy (12, 13). In contrast, pro-inflammatory cytokines seems to serve as biomarkers of persistent arthralgia (14, 15).

The aim of this study was to describe chemokine and cytokine profile during the acute phase of CHIKV infection, characterize the kinetics of serum chemokines and cytokines during acute disease, and evaluate whether specific serum biomarkers can predict the risk of development of chronic arthralgia.

## Materials and Methods

### Patients and Study Design

This study was carried out as part of an enhanced surveillance investigation designed to monitor arboviral infections among acute febrile patients at an emergency health center in Salvador, Brazil, between September 2014 and July 2016. The surveillance was previously described (6, 16). Briefly, a study team enrolled patients with at least 6 months of age, with residence in Salvador, and with reported or measured fever of ≤7 days of duration at the date of attendance. We performed blood collection and interviewed the patients to obtain demographic, epidemiological, and clinical data. All interviews were entered into a Research Electronic Data Capture (REDCap) digital database (17). Acute-phase (≤7 days after symptoms onset) and convalescent-phase (15-40 days after symptoms onset) blood samples were collected, centrifuged and sera were frozen at -80°C and -20°C until molecular and serological analyses, respectively. Between November 2016 and March 2017, telephone follow-up was performed for all patients who had laboratory evidence of CHIKV infection to obtain self-reported data on disease resolution or progression and on duration of arthralgia (10).

Molecular diagnosis of CHIKV was carried by conventional RT-PCR according to specific protocols (18–20). The viral RNA was extracted from the patients’ samples using Maxwell^®^ 16 Viral Total Nucleic Acid Purification Kit (Promega, Madison, USA) or QIAmp^®^ Viral RNA mini kit (Qiagen, Hilden, Germany) assays according to the manufacturer’s specifications. Detection of specific anti-CHIKV IgM antibodies was performed using the CHIKjj Detect ™ IgM ELISA kits (InBios International, Seattle, USA).

The enhanced surveillance enrolled 948 acute febrile illness patients with at least 1 sample available for laboratory testing. Amongst them, 265 (28%) had molecular or serological evidence of CHIKV infection. Of the 683 CHIKV negative patients, 115 (17%) were positive for dengue virus (DENV) (102 patients) or Zika virus (ZIKV) (13 patients) by conventional RT-PCR (protocols of Lanciotti et al., 1992 and Balm et al., 2012), or by detection of DENV IgM antibody (Dengue IgM Capture ELISA, Panbio Diagnostics, Brisbane, Australia) or DENV NS1 antigen (Dengue Early ELISA kit, Panbio Diagnostics, Brisbane, Australia). The remaining 568 (83%) patients did not have any laboratory evidence for CHIKV, DENV or ZIKV infection and thus were deemed to have other acute febrile diseases (OAFD) of non-arboviral etiology.

Chemokines and cytokines were measured in -80°C acute-phase serum samples from 253 of the 265 patients with laboratory evidence of CHIKV infection; 12 patients were not tested due to the insufficient volume of serum. These 253 chikungunya patients were further categorized into subgroups according to: *i)* method of chikungunya diagnosis (solely based on IgM detection on acute-phase sample (n=81) *vs* based on RT-PCR on acute-phase sample or by IgM seroconversion between paired samples (n=172); *ii)* number of days post symptoms onset (DPSO) at the enrollment and collection of acute-phase blood (1 DPSO (n=109), *vs* 2-3 DPSO (n=83), *vs* ≥ 4 DPSO (n=61)); and *iii)* duration of the arthralgia (≤3 months (n=84) *vs >*3 months, which was used to define patients with chronic arthralgia (n=62); follow-up was not attained for 107 patients).

Chemokine and cytokine levels from patients with CHIKV infection were compared with those from two control groups. The first comparison group consisted of patients randomly selected from those enrolled by the enhanced surveillance study, whose results for CHIKV, DENV and ZIKV were negative, and whose acute-phase sera were available at -80°C (defined as patients with OAFD; n=81). The second comparison group comprised healthy controls (HC) (n=15). Samples from HC subjects were negative by CHIKV qRT-PCR Assay (21), DENV IgM ELISA (Panbio Diagnostics, Brisbane, Australia) and CHIKV IgM and IgG ELISA (Euroimmun, Lubeck, Germany).

This study was approved by the Research Ethics Committee of Gonçalo Moniz Research Center, Oswaldo Cruz Foundation, Salvador, Brazil (CAAE: 90874818.5.0000.0040).

### Serum Biomarkers Measurement

Serum cytokines and chemokines levels were measured by flow cytometry, using commercially available BD Cytometric Bead Array™ (CBA) kits. Human Th1, Th2, Th17, Human Inflammatory Cytokine and Human Chemokine (BD Biosciences, San Diego, CA, USA) kits were used according to the manufacturer’s instructions using BD FACSArray™ equipment (BD Biosciences, San Jose, CA, USA). A total of 14 serum biomarkers where analyzed: CXCL8, CXCL9, CCL2, CCL5, CXCL10, IL-1β, IL-6, IL-12, TNF, INF-γ, IL-17, IL-2, IL-4 and IL-10. Data were analyzed using FCAP Array version 3.0 (BD Biosciences, USA) and the results were expressed in pg/mL for chemokines and in mean fluorescence intensity (MFI) for cytokines due to their low limit of detection in the CBA tests.

### Statistical Analysis

One-way ANOVA followed by Tuckey test or Kruskal Wallis followed by Dunns Test were used for multiple comparisons amongst subgroups. Student t-Test or Mann-Whitney Test were used for comparison between two groups. Chi-square test was used for comparative analysis of categorical variables. In all cases, statistically significant differences were set at P <0.05.

Among patients who had information for duration of arthralgia, additional comparisons of chemokines and cytokines concentrations were performed using bivariate Poisson regression models. Biomarkers presenting P value <0.20 and variables for clinical characteristics presumed to influence arthralgia duration, such as age, gender, or the biomarker level, such as days of symptoms at sample collection, were entered into a backward multivariable Poisson regression model. Results were expressed as risk ratios (RR) for arthralgia lasting > 3 months and 95% Confidence Intervals (95% CI). The final model retained variables associated with duration of arthralgia (P <0.05).

Data were analyzed using GraphPad Prism version 5.0 (GraphPad Software Inc., San Diego, CA) and STATA software version 13 (StataCorp LP, College Station, TX, USA). Graphics were prepared using GraphPad Prism version 5.0 (GraphPad Software Inc., San Diego, CA) and Excel 2007 for Windows (Microsoft, USA).

## Results

### Participants’ Characteristics

No statistically significant difference in age median or sex was observed between patients with chikungunya and patients with OAFD. However, comparing the frequency of individuals by age categories, we found that patients with OAFD had more similar proportion of cases by age groups, while patients with chikungunya had a higher frequency of young and adult individuals (15 to 44 years) (**Table 1**). Patients with chikungunya had significantly higher frequencies of myalgia, arthralgia (including polyarthralgia and symmetric arthralgia), and swollen joints in the acute phase of the disease (P <0.05). Conversely, patients with OAFD had higher frequencies of cough and sore throat (P <0.05). In addition, patients with OAFD had longer duration of symptoms when presenting for initial care (median: 3 days; interquartile range (IQR): 2-4) compared to patients with chikungunya (median: 2 days; IQR: 1-3) (P <0.05). Of the 81 patients with OAFD, 37 (45%) had a clinical suspicion recorded in the medical chart. The most common suspicions were arboviral infection (14; 17%), unspecified viral infection (11; 14%), and respiratory infections (9; 11%).

**Table 1:** Acute-phase clinical characteristics of patients with chikungunya virus (CHIKV) infection compared to that of patients with other acute febrile diseases (OAFD), and according to duration of arthralgia after CHIKV infection, Salvador, Brazil, 2014-2016.

Characteristics	Chikungunya and other acute febrile diseases		*P values*	Chikungunya cases according to duration of arthralgia ***			*P values*
	CHIKD (n = 253)	OAFD (n = 81)		≤ 3 months (n = 84)		> 3 months (n = 62)	
	% (numerator/denominator) or Median (IQR)			% (numerator/denominator) or Median (IQR)			
Sex			0.99				<0.01
Female	51 (128/253)	51 (41/81)		35 (35/84)	68 (42/62)		
Male	49 (125/253)	49 (40/81)		65 (49/84)	32 (20/62)		
Age, in years	34 (22 - 44)	30 (14 - 50)	0.36	32.5 (21-42)	39 (32-49)		<0.01
<15	12 (30/253)	27 (22/81)	<0.01	13 (11/84)	5 (3/62)		0.03
≥15 and ≤29	30 (77/253)	22 (18/81)		33 (28/84)	16 (10/62)		
≥30 and ≤44	33 (82/253)	17 (14/81)		30 (25/84)	44 (27/62)		
≥45 and ≤59	16 (40/253)	25 (20/81)		14 (12/84)	19 (12/62)		
≥60	9 (23/253)	9 (7/81)		10 (8/84)	16 (10/62)		
Days post symptoms onset	2 (1-3)	3 (2-4)	<0.01	2(1-3)	2 (1-4)		0.22
Myalgia	92 (234/253)	83 (67/81)	0.02	90 (76/84)	93 (58/62)		0.50
Arthralgia	88 (224/253)	63 (51/81)	<0.01	84 (71/84)	95 (59/62)		0.04
Polyarthralgia*	81 (205/253)	49 (40/81)	<0.01	76 (64/84)	90 (56/62)		0.02
Symmetric Arthralgia**	82 (207/253)	58 (47/81)	<0.01	73 (62/84)	95 (59/62)		<0.01
Headache	92 (234/253)	94 (76/81)	0.68	91 (77/84)	92 (57/62)		0.95
Retro-orbital pain	70 (176/252)	71 (57/80)	0.81	64 (54/84)	77 (48/62)		0.08
Swollen Joints	41 (105/253)	13 (11/81)	<0.01	28 (24/84)	58 (36/62)		<0.01
Rash	32 (81/253)	33 (27/81)	0.85	24 (20/84)	38 (23/62)		0.07
Conjunctival Hyperemia	48 (98/204)	35 (14/40)	0.13	56 (37/66)	50 (26/52)		0.51
Cough	34 (89/253)	62 (51/81)	<0.01	33 (28/84)	32 (20/62)		0.89
Sore throat	27 (69/253)	54 (44/81)	<0.01	26 (22/84)	24 (15/62)		0.78
Diarrhea	17 (44/253)	26 (21/81)	0.09	12 (10/84)	22 (14/62)		0.08
Nausea/Vomiting	23 (59/253)	27 (21/80)	0.60	18 (15/83)	26 (16/62)		0.26

CHIKD, patients with acute CHIKV infection detected by IgM ELISA and/or RT-PCR for CHIKV;

OAFD, patients with other acute febrile disease (negative by IgM ELISA and RT-PCR for CHIKV and DENV and negative by RT-PCR for ZIKV);

IQR, Interquartile range;

* > 1 joint involved;

**At least one pair of joints involved;

***Duration of arthralgia determined by telephone follow-up. Of the 253 patients with chikungunya, follow-up was completed and data on arthralgia duration obtained for 146 patients.

Patients with chikungunya that developed chronic arthralgia were older at the acute-phase of the disease (median: 39 years; IQR 32-49) than those that did not (median age 32.5 years; IQR: 21-42) (P <0.01) and most were women (68% *vs* 42%, respectively, P <0.01) (**Table 1**). In addition, patients with chronic arthralgia had significantly higher frequency of swollen joints, arthralgia (symmetric and polyarticular) at the acute phase of chikungunya compared to those that recovered from arthralgia in ≤3 months. Chikungunya patients diagnosed by detection of anti-CHIKV IgM antibodies in the acute-phase serum had a higher interval between symptoms onset and medical care (median: 4 days; IQR: 2-6) compared to patients diagnosed by RT-PCR or IgM seroconversion (median: 1 day; IQR: 1-3) (P <0.05) (**Supplementary Table 1**). Additionally, the group of patients diagnosed by detection of CHIKV IgM antibodies in the acute-phase serum more frequently had rash, cough, sore throat, and diarrhea, but less frequently had arthralgia (P <0.05).

### Chemokines and Cytokines Profile in Patients With Acute Chikungunya

**Figure 1:**
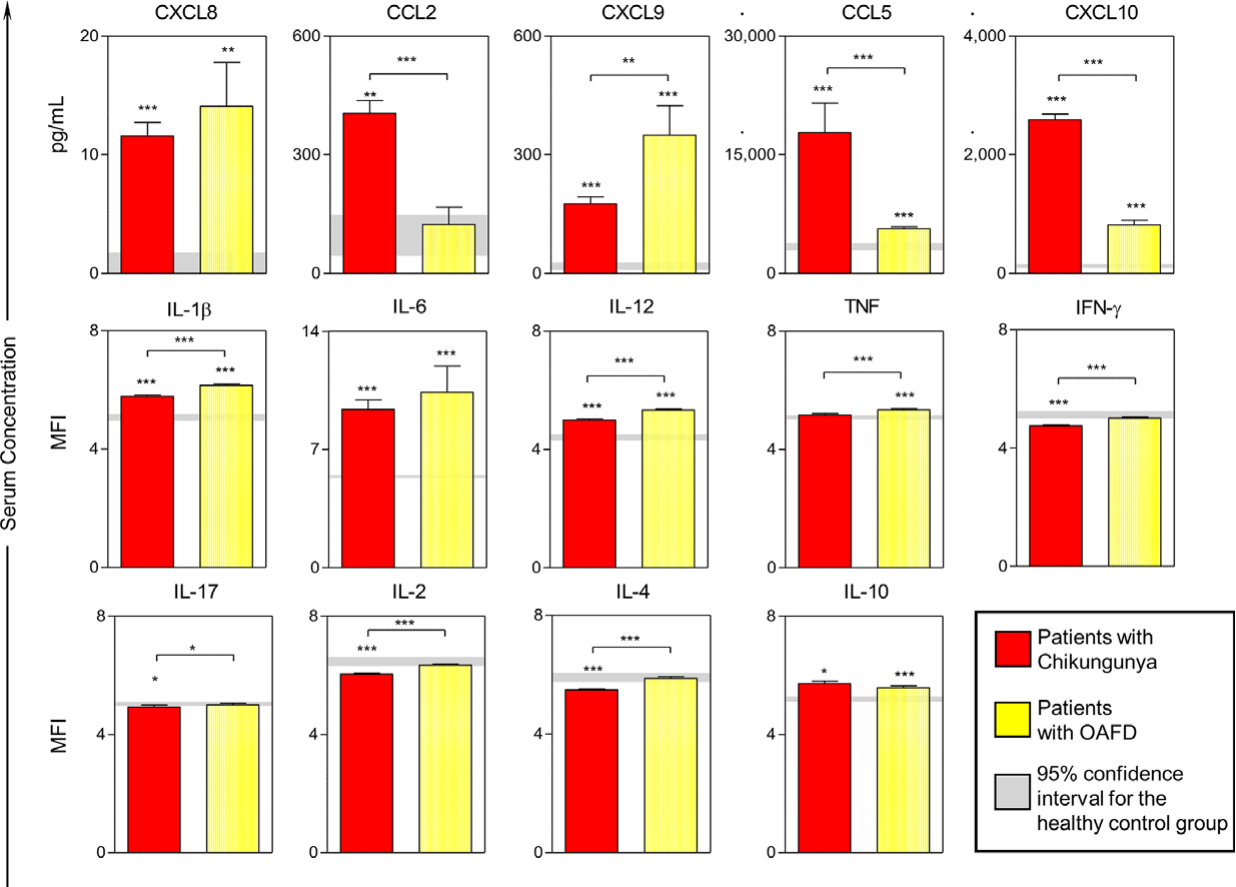
Serum levels of chemokines and cytokines in patients with acute chikungunya virus (CHIKV) infection and other acute febrile diseases. A total of 14 soluble biomarkers (CXCL8, CXCL9, CCL2, CCL5, CXCL10, IL-1β, IL-6, IL-12, TNF, INF-γ, IL-17, IL-2, IL-4 and IL-10) were measured in serum samples from patients with acute CHIKV infection and other acute febrile diseases (OAFD) as well as healthy controls (HC). Results are expressed in pg/mL for chemokines and in mean fluorescence intensity for cytokines. Data are presented as mean values; the error bar around the means represent standard error of the mean. The gray lines represent the 95% confidence intervals for the mean values obtained for the healthy controls. Statistically significant differences were identified by asterisks for comparisons with HC and by asterisks above connecting lines for differences between patients with chikungunya and OAFD (*p < 0.05; **p < 0.005; ***p < 0.001).

Compared to the HC group, patients with chikungunya had significantly higher levels for most chemokines and cytokines evaluated, except for TNF (for which no difference was observed) and IFN-γ, IL-17, IL-2, and IL-4 (for which levels were significantly lower than that for HC) (**Figure 1**). Compared to patients with OAFD, patients with chikungunya had statistically higher levels of chemokines CCL2, CCL5, and CXCL10 and statistically lower levels of CXCL9, IL-1β, IL-12, TNF, IFN-γ, IL-17, IL-2, and IL-4.

### Chemokines and Cytokines Profile According to Subgroups of Patients With Acute Chikungunya

**Figure 2:**
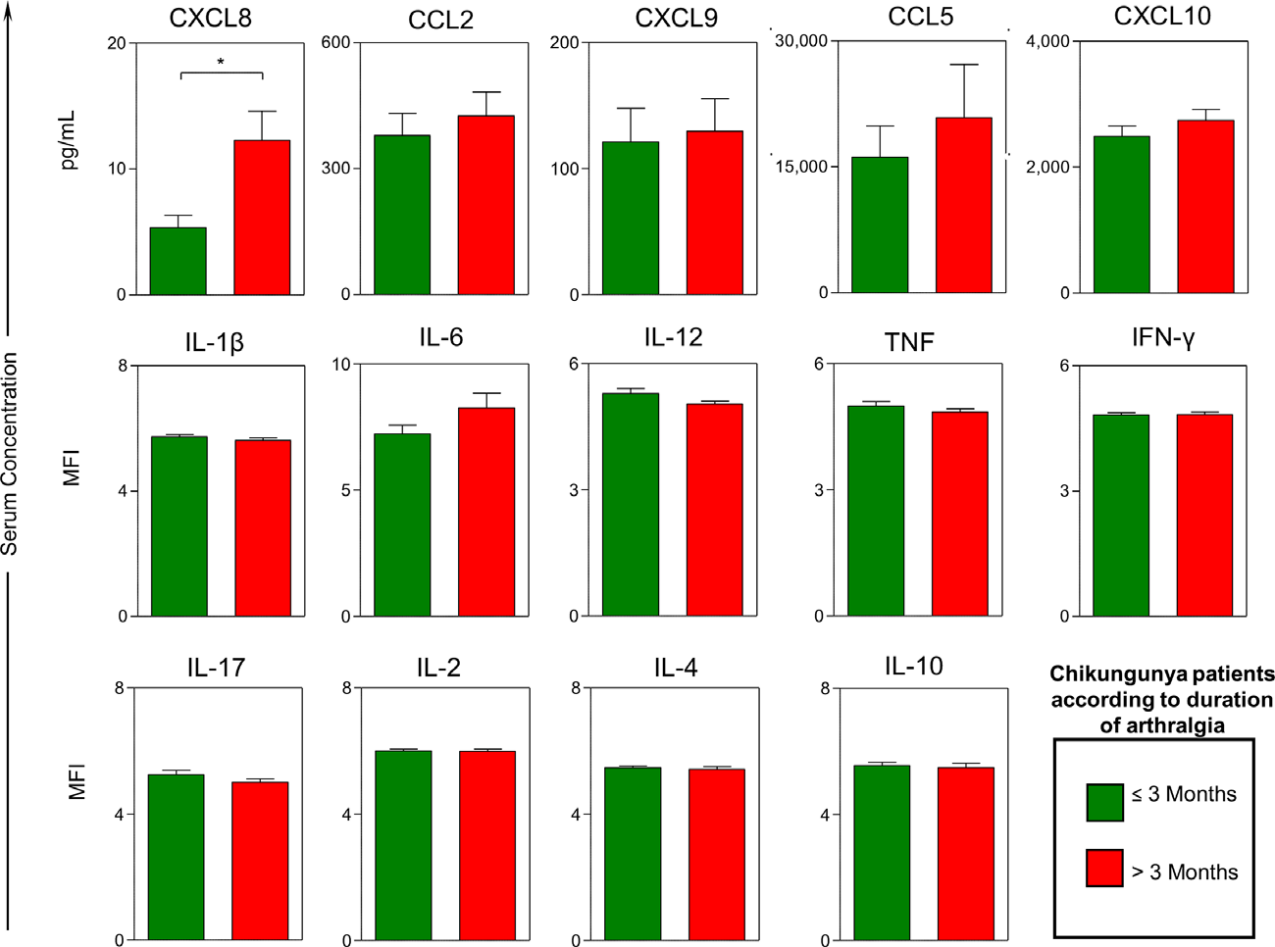
Serum levels of chemokines and cytokines in patients with acute chikungunya virus (CHIKV) infection according to the duration of arthralgia. Quantitative analysis of chemokines and cytokines was carried out by BD Cytometric Bead Array™ (CBA) as described in Methods. A total of 14 soluble biomarkers (CXCL8, CXCL9, CCL2, CCL5, CXCL10, IL-1β, IL-6, IL-12, TNF, INF-γ, IL-17, IL-2, IL-4 and IL-10) were measured in serum samples from patients with acute CHIKV infection categorized according to the duration of arthralgia (≤ 3 months and > 3 months). Results are expressed in pg/mL for chemokines and in mean fluorescence intensity for cytokines. Data are presented as mean values; the error bar around the means represent standard error of the mean. Statistically significant differences were identified by asterisks above connecting lines for differences between patients with arthralgia lasting ≤ 3 months and > 3 months after CHIKV infection (*p < 0.05).

Levels of CXCL8 were significantly higher in patients with chikungunya whose arthralgia lasted >3 months compared to those whose arthralgia lasted ≤3 months (**Figure 2**). Levels for all the other chemokines and cytokines did not significantly differ between these groups. Bivariate Poisson regression analysis found that age, female sex, days of symptoms, higher levels of CXCL8, IL-6 and lower levels of TNF were associated with arthralgia lasting >3 months (**Table 2**). However, only CXCL8 (RR=1.04; 95% CI: 1.01-1.08) and female sex (RR=3.24; 95% CI: 1.51-6.94) remained associated with chronic arthralgia in a multivariable Poisson regression model that retained adjustment for age and days of symptoms at acute-phase sample collection (**Table 2**).

**Table 2:** Predictors of chronic arthralgia among patients with chikungunya virus acute infection, Salvador, Brazil, 2014-2016.

	Risk Ratio for arthralgia lasting > 3 months (95% CI)			
Factor	Bivariable model	*p values*	Multivariable model	*p values*
**Demographics and clinical characteristics**				
**Female sex**	**3.01 (1.51-5.97)**	**<0.01**	**3.24 (1.51-6.94)**	**<0.01**
Age	1.03 (1.00-1.05)	<0.01	1.01 (0.99-1.04)	0.13
Days of symptoms	1.03 (1.01-1.05)	<0.01	1.07 (0.97-1.19)	0.13
**Serum chemokines and cytokines**				
**CXCL8**	**1.01 (1.00-1.01)**	**<0.01**	**1.04 (1.01-1.08)**	**<0.01**
CCL2	1.00 (0.99-1.00)	0.17	*	*
CXCL9	1.00 (0.99-1.00)	0.81	*	*
CCL5	0.99 (0.99-1.00)	0.36	*	*
CXCL10	1.00 (0.99-1.00)	0.28	*	*
IL-1β	0.86 (0.64-1.15)	0.31	*	*
IL-6	1.03 (0.99-1.06)	0.03	*	*
TNF	0.78 (0.61-0.91)	0.04	*	*
IL-12	0.85 (0.66-1.10)	0.23	*	*
IFN-γ	1.01 (0.67-1.53)	0.92	*	*
IL-17	0.86 (0.70-1.05)	0.14	*	*
IL-4	0.93 (0.65-1.31)	0.67	*	*
IL-10	0.98 (0.81-1.20)	0.91	*	*
IL-2	0.95 (0.65-1.39)	0.82	*	*

*Variables not entered or retained into the final Poisson multivariable model. The final model was initially built by inclusion of variables associated at P<0.20, followed by backward removal of variables not associated at P<0.05, excepted by age and days of symptoms that were retained in the model for adjustment. Bold font indicates variables associated with chronic arthralgia in the final model. Risk Ratio (RR) indicates increase in risk of chronic arthralgia per one unit increase in the exposure variable (which for chemokines are expressed in pg/mL and for cytokines are expressed in median fluorescence intensity). Except for female sex, which is a categorical variable and the observed RR was estimated compared to men, all other variables are quantitative.

Compared to the subgroup of patients with CHIKV IgM antibodies detected at the acute-phase serum, the subgroup of patients with diagnosis by RT-PCR or IgM seroconversion had significantly higher levels of CXCL8, CCL2, CXCL10, and IL-6 (**Supplementary Figure 1**). In contrast, they had significantly lower levels of IFN-y, IL-2, and IL-4. Because of this difference, we performed subgroup analysis on the 100 chikungunya patients diagnosed by RT-PCR or IgM seroconversion who completed follow-up (45 with arthralgia lasting >3 months and 55 with arthralgia lasting ≤3 months) and found that CXCL8 (RR=1.01; 95% CI: 1.00-1.01) and IL-6 (RR=1.03; 95% CI: 1.00-1.06) levels were associated with chronic arthralgia in bivariate Poisson models.

### Kinetic Profile of Chemokines and Cytokines in Patients With Acute Chikungunya

**Figure 3:**
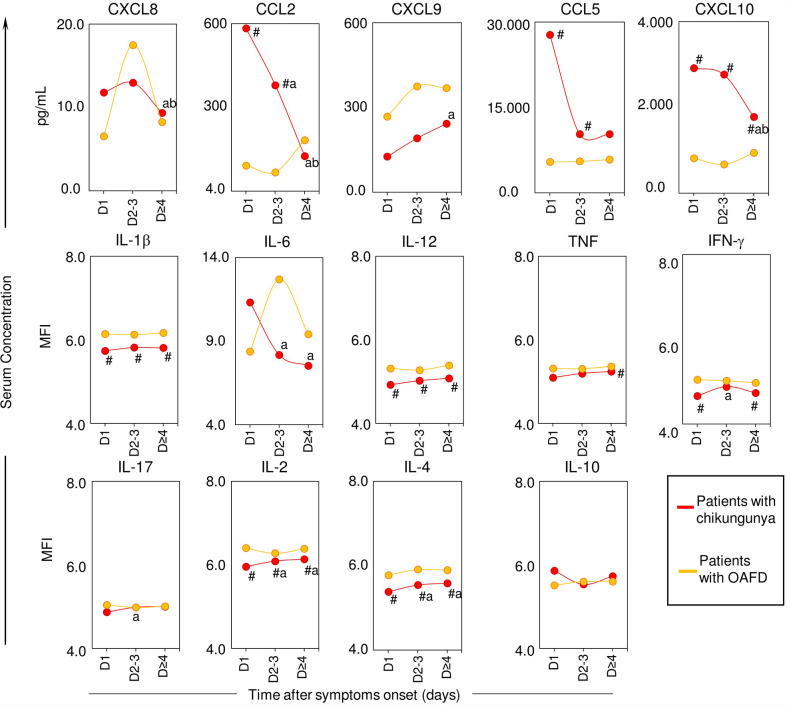
Kinetic profile of serum chemokines and cytokines in patients with acute chikungunya virus (CHIKV) infection and other acute febrile diseases. A total of 14 soluble biomarkers (CXCL8, CXCL9, CCL2, CCL5, CXCL10, IL-1b, IL-6, IL-12, TNF, INF-g, IL-17, IL-2, IL-4 and IL-10) were measured in serum samples from patients with chikungunya and patients with other acute febrile diseases (OAFD) at distinct times after symptoms onset (D1, D2-3 and D≥4) as well as healthy controls. Data are presented as mean values and significant differences at p<0.05 by "#" for differences between chikungunya and OAFD and letters “a” and “b” for comparisons with D1 and D2-3, respectively. The results are expressed in pg/mL for chemokines and in mean fluorescence intensity for cytokines.

To describe the kinetic profile of chemokines and cytokines in patients with chikungunya, we observed their mean levels according to the interval between symptoms onset and blood sample collection (1, 2-3, or ≥ 4 DPSO), and compared these levels with those observed of patients with OAFD (**Figure 3**). For both chikungunya and OAFD patients, the mean levels of IL-1β, IL-12, TNF, IFN-γ, IL-2 and IL-4 did not substantially fluctuate during the evaluated period. However, patients with chikungunya commonly had a lower magnitude of these biomarkers than patients with OAFD (**Figure 3**). On the other hand, patients with chikungunya presented a decreasing trend in the levels of CXCL8, CCL2, CCL5, CXCL10, and IL-6 after 1 or 2-3 DPSO, but patients with OAFD did not follow this pattern. Both patients with chikungunya and with OAFD had an increasing trend in the levels of CXCL9 over time.

## Discussion

Our analysis of the serum biomarker profile of patients with acute CHIKV infection showed increased levels of pro-inflammatory Th1 chemokines (CCL5, CCL2, CXCL10) and reduced levels of IL-2, TNF and mediators of Th2 and regulatory responses (IL-12, IL-4 and IL-10) compared to the control group of patients with OAFD. We also found that chikungunya patients who developed chronic arthralgia had higher levels of CXCL8 in the acute phase of the disease compared to those who did not, even after adjusting for age, sex and number of days of symptoms at the time of sample collection. This finding suggests that CXCL8 may have an inflammatory role during the acute-phase of the disease, which may contribute to the development of chronic arthralgia.

Intense arthralgia is one of the most striking features of chikungunya and it may persist for several months (22). In this study, 88% and 63% of the patients with chikungunya and with OAFD, respectively, reported arthralgia during the acute phase of the disease. In contrast, patients with OAFD had higher frequencies of cough and sore throat, clinical manifestations that suggest that they frequently had an influenza-like illnesses (23). Patients with chikungunya also sought medical care earlier than patients with OAFD, which may be due to the intensity of pain caused by CHIKV infection.

It is possible that some of the differences in the clinical manifestations observed between patients with acute chikungunya and those with OAFD were due to a distinct profile of chemokines and cytokines. Our analysis showed that patients with acute chikungunya had significantly higher levels of the serum chemokines CCL2, CCL5, and CXCL10 compared to patients with OAFD. CCL2 is a potent monocyte-attracting chemokine (24) and CCL5 greatly contributes to the recruitment of blood monocytes, eosinophils, basophils, monocytes, effector memory T cells, B cells, NK cells, and immature dendritic cells into inflammation sites and high levels of both had been previously described in patients with CHIKV infection (25).

We had previously reported that 42.5% of the 153 chikungunya patients detected during our surveillance for acute febrile illness that were followed by telephone calls developed chronic arthralgia that lasted >3 months (10). In this subsample of 146 chikungunya patients that were followed and had available sera for cytokine and chemokine testing, the frequency of chronic arthralgia was exactly the same (42.5%). This rate is within the rate of chronic joint manifestations after 3 months of CHIKV infection, which has been estimated at 43% (95% CI, 35-52%) (26). Methodological issues, such as sample size, duration of follow-up, the strategy used to determine the outcome (by telephone or medical evaluation), and data collection methods may partly explain the variability between the results (27).

In this study, we found female sex as a risk factor for the development of chronic arthralgia. Previous studies have found similar findings (28–31). In general, females tend to develop a greater proinflammatory response compared to males and, resulting in sex-specific outcomes from infectious and autoimmune diseases (32, 33). Greater proinflammatory response observed in woman may cause more severe symptomatology in CHIKV infection in comparison with man (34).

In addition, we found that higher levels of CXCL8 in acute-phase samples of chikungunya patients was significantly associated with development of arthralgia that last >3 months. This is consistent with other studies that reported an increase in circulating levels of CXCL8 in patients with CHIKV infection (35). This specific chemokine is secreted by activated macrophages mainly during the innate immune response and promotes the recruitment of neutrophils to the inflammatory site (36). Reddy et al. (25) also found that chikungunya patients who had chronic arthralgia had elevated levels of CXCL8 in the second week after symptom onset. Several other studies have reported elevated serum CXCL8 levels in the acute phase compared to healthy controls (35, 37, 38), and an even greater increase in levels of this chemokine in the chronic phase of the disease compared to the acute phase (39). Furthermore, CXCL8 has also been detected in serum (40) and synovial fluid (14, 41) of patients with chronic arthralgia after CHIKV infection. On the other hand, some studies found that reduced levels of CXCL8 were associated with increased intensity of pain (42) and chronic arthralgia (43). Yet, in this latest study, CXCL8 values were also increased in the acute phase of the disease compared to healthy controls. Many reasons can lead to differences between cytokine and chemokine levels in patients from different studies. Results may vary depending on study design, cohort size, disease stage, number of days of symptoms at the time of sample collection, genetic history, among others (44). However, the results found in this study are in agreement with most studies that previously performed this type of assessment.

Recent work showed that neutrophil extracellular traps effectively control CHIKV infection *in vitro* human cells model and in challenged mice, reinforcing that Th1 cells and mediators early recruited to the infection site could be able to control CHIKV replication and dissemination (45). It has also been suggested that occurrence of joint pain and inflammation following CHIKV infection may be associated with dysregulation of the immune response in both the acute and convalescent phases, with an increase in Th1 pro-inflammatory cytokines and decrease in Th2 effectors cytokines (14, 38), which suggests that the Th1 response could either fight CHIKV infection and resolve the symptoms or promote more inflammation and pain. Therefore, it is important to note that the correlation between joint inflammation and a state of systemic activation, as demonstrated by the presence of inflammation markers in plasma, such as chemokines and cytokines, remains uncertain. It is not clear whether the chronic arthralgia resulting from CHIKV infection is due to the viral presence in joints and tissues, whether these events are due to the host’s immune response to infection or even an association of these events. There may be other factors that interfere with the virus-host interaction, such as genetic background (e.g., polymorphism, human leukocyte antigen), which can trigger and direct the immune response and, consequently, influence the patient’s outcome.

As expected, patients who sought medical care later (median 4 days of symptoms) were confirmed by CHIKV IgM antibodies, whereas patients that sought medical care earlier (median 1 day of symptom) were confirmed by RT-PCR/IgM seroconversion. Thus, patients diagnosed by RT-PCR or by IgM seroconversion had levels of chemokines and cytokines compatible with those observed for patients diagnosed earlier in the course of the acute disease (in the first three days after the onset of symptoms); for example, they had higher levels of IL-6, CXCL8, CCL2, CXCL10, which are known to recruit Th1 cells levels. In contrast, patients diagnosed by IgM detection on the acute-phase sample had chemokines and cytokines levels compatible with those observed for patients detected later on the course of the acute disease, such as higher levels of IL-2 and IL-4.

Our study has some limitations. First, we cannot rule out that some of our chikungunya patients diagnosed by the presence of IgM in the acute-phase sample were actually seeking medical care due to another disease and maintained the CHIKV IgM from a previous infection. Our patients diagnosed by detection of CHIKV IgM in the acute-phase sample had higher frequencies of rash, cough, sore throat, and diarrhea (P <0.05) compared to those diagnosed by RT-PCR or IgM seroconversion, a profile similar to that of patients with OAFD. Furthermore, a recent study (46) showed that a small fraction of patients with chikungunya can maintain detectable levels of CHIKV IgM antibodies for long periods; 7 (12%) of 57 patients maintained detectable IgM antibodies after 15 months of follow-up. Other authors had already reported similar findings regarding persistence of CHIKV IgM, ranging from 13.2% (30/227) after 12-13 months to 76% (16/21) after 10 months (47, 48). Based on this, they recommend that, whenever possible, the laboratory diagnosis of CHIKV infection should not rely only on serological results, but rather be based on molecular biology tests that can directly detect the presence of the virus. Although not all of our chikungunya patients had a positive RT-PCR result, 68% of them were diagnosed by RT-PCR or IgM seroconversion, indicating that the majority undoubtedly had an acute infection. In addition, subgroup bivariate analysis performed on the chikungunya cases diagnosed by RT-PCR or IgM seroconversion confirmed that CXCL8 and IL-6 were positively associated with chronic arthralgia.

Second, we were not able to determine the etiological diagnosis for the comparison group of patients with OAFD, but all of them had the diagnosis of chikungunya, as well as dengue and Zika, discarded, and their clinical picture suggest that an acute respiratory illness was the most probable etiology for them. Another limitation of our study includes the low dosage levels of certain cytokines (CBA Human Th1, Th2, Th17 and Inflammation), which led us to choose the use of MFI, instead of pg/mL, to present the results for them. Although other studies have used this unit of measurement in cytokines analysis, we acknowledge that this type of measurement may not be ideal for evaluating biomarkers. Lastly, we detected persistence of chronic arthralgia through telephone follow-up, which prevented us from carrying out a detailed clinical assessment. Thus, we cannot completely rule out the possibility of patients’ misclassification.

Due to the spread of CHIKV and the occurrence of large epidemics in all tropical and subtropical regions of the world, a better understanding of the factors that influence the onset of chronic arthralgia after infection is critical to guide the development of preventive and therapeutical approaches targeting chikungunya joint pain. Nevertheless, the precise functional role of the immune response and its mediators in CHIKV-infected individuals remains a challenge. This study contributed to clarify the relationship between the initial immune response after CHIKV infection and the clinical progression of the disease. In addition, our findings highlighted that CXCL8 probably plays a key role in the immunopathogenesis of chronic arthralgia. However, the precise biological implication associated with the CXCL8 profile observed in our study remains to be elucidated and it should be considered in future research on biomarkers, drug targets, and therapeutic monitoring of chikungunya patients. Further investigations into the immunopathogenesis of chikungunya and to evaluate pharmacological and non-pharmacological approaches aiming to improve the quality of life of patients with chikungunya are needed.

## Data Availability Statement

The raw data supporting the conclusions of this article will be made available by the authors, without undue reservation.

## Ethics Statement

This study was reviewed and approved by the Ethics Research Committee of the Gonçalo Moniz Institute, Oswaldo Cruz Foundation (CAAE 55904616.4.0000.0040). Before enrollment into this study, all subjects ≥18 years provided written informed consent and all subjects aged 7–17 years provided written informed assent. Parents or legal guardians also provided written informed consent for inclusion of patients <18 years of age.

## Author Contributions

Designing research study: GR, MR. Conducting experiments: LJ-N, CC, LT, PM. Acquiring data: LJ-N, LT, PM, MK, MS, RA. Analyzing data: LJ-N, JF, AC-A, OM-F. Advisory Medical Committee: GR, MR. Writing the manuscript: LJ-N, GR, MR, OM-F. Revising the manuscript: LJ-N, JF, AC-A, GR, MR, MP, CC, MS, MK, RA, LT, GC, PM, OM-F. All authors contributed to the article and approved the submitted version.

## Funding

This work was supported by the Brazilian National Council for Scientific and Technological Development under Grants 400830/2013-2 and 440891/2016-7 to GR, and scholarships to LT, MR, and GR; the Bahia Foundation for Research Support under Grants PET0026/2013, PP0044/2016, and PET0022/2016 to GR, and scholarship to MS; the Coordination for the Improvement of Higher Education Personnel, Brazilian Ministry of Education under grant 88881.130749/2016-01 to GR; the Department of Science and Technology, Secretariat of Science, Technology and Strategic Inputs, Brazilian Ministry of Health; the Federal University of Bahia; the Oswaldo Cruz Foundation; and the REPLICK (Clinical and Applied Research in Chikungunya). OM-F is a research fellow from FAPEAM (PVN-II, PRÓ-ESTADO Program #005/2019). The funders had no role in study design, data collection and analysis, decision to publish, or preparation of the manuscript.

## Conflict of Interest

The authors declare that the research was conducted in the absence of any commercial or financial relationships that could be construed as a potential conflict of interest.

## Publisher’s Note

All claims expressed in this article are solely those of the authors and do not necessarily represent those of their affiliated organizations, or those of the publisher, the editors and the reviewers. Any product that may be evaluated in this article, or claim that may be made by its manufacturer, is not guaranteed or endorsed by the publisher.
