# Supplementary material for: Acute-Phase Levels of CXCL8 as Risk Factor for Chronic Arthralgia Following Chikungunya Virus Infection
Source: Front Immunol. 2021 Oct 1;12:744183. doi: 10.3389/fimmu.2021.744183 (PMC8517435; doi:10.3389/fimmu.2021.744183)
Supplement: Supplementary file 1 [file DataSheet_1.pdf]

Supplementary Material

Supplementary Table 1. Clinical characterization of patients with Chikungunya according to method of laboratorial diagnosis, Brazil, 2014-2016

**Parameters**	**RT-PCR / Seroconversion** (n=172)	**IgM** (n=81)	*p values*
**Female sex**	51 (88/172)	49 (40/81)	0.06
**Age (years)**	32 (20 – 43.5)	35 (25-46)	0.18
**Days post symptoms onset**	1 (1-3)	4 (2-6)	<0.01
**Myalgia**	93 (161/172)	90 (73/81)	0.32
**Arthralgia**	94 (163/172)	75 (61/81)	<0.01
**Polyarthralgia***	87 (150/172)	67 (55/81)	<0.01
**Symmetric Arthralgia****	86 (148/172)	72 (59/81)	0.01
**Headache**	92 (159/171)	92 (75/81)	0.96
**Retro-orbital pain**	70 (120/172)	69 (56/81)	0.86
**Swollen Joints**	43 (74/172)	38 (21/81)	0.47
**Rash**	25 (43/172)	46 (38/81)	<0.01
**Conjunctival Hyperemia**	50 (78/155)	40 (20/49)	0.24
**Cough**	27 (47/172)	51 (42/81)	<0.01
**Sore throat**	17 (30/172)	48 (39/81)	<0.01
**Diarrhea**	13 (23/172)	25 (21/81)	0.01
**Nausea/Vomiting**	23 (40/172)	23 (19/81)	0.56

* > 1 joint envolved; ** At least one pair of joints with arthralgia; RT-PCR/Seroconversion group comprises patients diagnosed based on RT-PCR on acute-phase sample or by IgM seroconversion between paired samples; IgM group comprises patients diagnosed solely based on IgM detection on acute-phase sample.

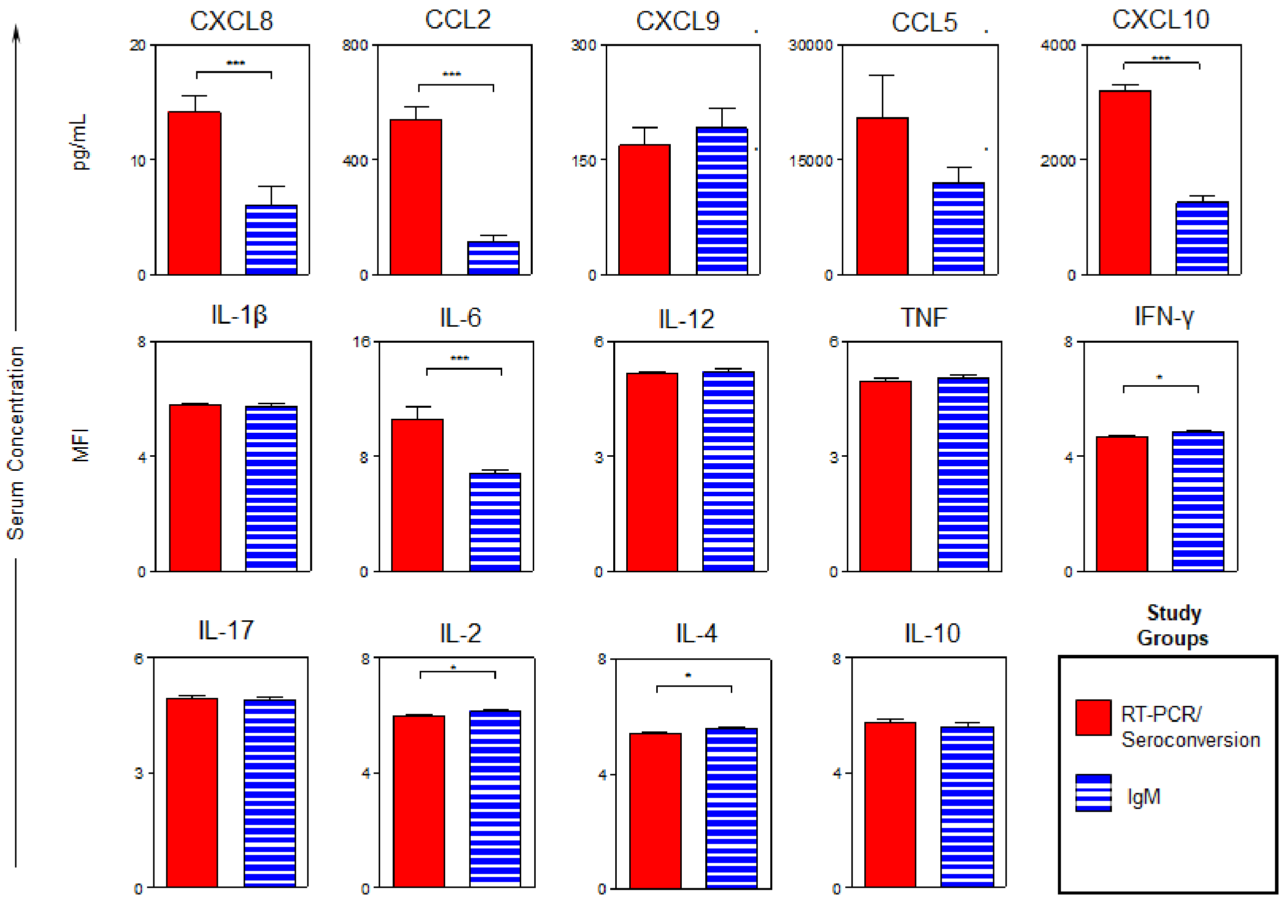

Supplementary Figure 1. Serum levels of chemokines and cytokines in patients with acute chikungunya virus (CHIKV) infection according to laboratorial diagnosis on the acute-phase sample. A total of 14 soluble biomarkers (CXCL8, CXCL9, CCL2, CCL5, CXCL10, IL-1β, IL-6, IL-12, TNF, INF-γ, IL-17, IL-2, IL-4 and IL-10) were measured in serum samples from patients with acute CHIKV infection categorized according to the CHIKV IgM and RT-PCR status: the IgM group comprises patients diagnosed solely based on IgM detection on acute-phase sample; the RT-PCR/Seroconversion group comprises patients diagnosed based on RT-PCR on acute-phase sample or by IgM seroconversion between paired samples. Results are expressed in pg/mL for chemokines and in mean fluorescence intensity for cytokines. Data are presented as mean values; the error bar around the means represents the standard error of the mean. Statistically significant differences were identified by asterisks above connecting lines for differences between patients with Chikungunya diagnosed solely based on IgM detection on acute-phase sample vs. Chikungunya diagnosed based on RT-PCR on acute-phase sample or by IgM seroconversion between paired samples (* $p<0.05$ and *** $p<0.001$).
